# Neural Processing of Emotional Musical and Nonmusical Stimuli in Depression

**DOI:** 10.1371/journal.pone.0156859

**Published:** 2016-06-10

**Authors:** Rebecca J. Lepping, Ruth Ann Atchley, Evangelia Chrysikou, Laura E. Martin, Alicia A. Clair, Rick E. Ingram, W. Kyle Simmons, Cary R. Savage

**Affiliations:** 1 Hoglund Brain Imaging Center, University of Kansas Medical Center, Kansas City, Kansas, United States of America; 2 Department of Psychology, University of Kansas, Lawrence, Kansas, United States of America; 3 Department of Preventive Medicine, University of Kansas Medical Center, Kansas City, Kansas, United States of America; 4 Department of Music Education and Music Therapy, University of Kansas, Lawrence, Kansas, United States of America; 5 Laureate Institute for Brain Research, Tulsa, Oklahoma, United States of America; 6 Faculty of Community Medicine, University of Tulsa, Tulsa, Oklahoma, United States of America; 7 Center for Health Behavior Neuroscience, University of Kansas Medical Center, Kansas City, Kansas, United States of America; 8 Department of Psychiatry, University of Kansas Medical Center, Kansas City, Kansas, United States of America; Max Planck Institute for Human Cognitive and Brain Sciences, GERMANY

## Abstract

**Background:**

Anterior cingulate cortex (ACC) and striatum are part of the emotional neural circuitry implicated in major depressive disorder (MDD). Music is often used for emotion regulation, and pleasurable music listening activates the dopaminergic system in the brain, including the ACC. The present study uses functional MRI (fMRI) and an emotional nonmusical and musical stimuli paradigm to examine how neural processing of emotionally provocative auditory stimuli is altered within the ACC and striatum in depression.

**Method:**

Nineteen MDD and 20 never-depressed (ND) control participants listened to standardized positive and negative emotional musical and nonmusical stimuli during fMRI scanning and gave subjective ratings of valence and arousal following scanning.

**Results:**

ND participants exhibited greater activation to positive versus negative stimuli in ventral ACC. When compared with ND participants, MDD participants showed a different pattern of activation in ACC. In the rostral part of the ACC, ND participants showed greater activation for positive information, while MDD participants showed greater activation to negative information. In dorsal ACC, the pattern of activation distinguished between the types of stimuli, with ND participants showing greater activation to music compared to nonmusical stimuli, while MDD participants showed greater activation to nonmusical stimuli, with the greatest response to negative nonmusical stimuli. No group differences were found in striatum.

**Conclusions:**

These results suggest that people with depression may process emotional auditory stimuli differently based on both the type of stimulation and the emotional content of that stimulation. This raises the possibility that music may be useful in retraining ACC function, potentially leading to more effective and targeted treatments.

## Introduction

Emotion regulation is a critical skill for emotional health and well-being [1]. Music is a powerful inducer of emotion, and people often report using music as a tool for regulating their emotional state [2–4]. Music is used for mood manipulations in clinical and laboratory settings [5–10]. Music is also used for mood change in the population generally, and as a coping strategy specifically in depression. College students diagnosed with depression report using music to reduce stress and anxiety [3]. Additionally, a European survey of public advice for the best self-help measures for dealing with depression placed listening to music near the top of the list, with 69% of respondents agreeing that they would recommend this as a useful self-help method. In this survey, 82% of respondents with depression who were already in treatment agreed that music was helpful in this regard [4]. Studies examining pleasurable musical experiences have associated enjoyment of music and activation in ventral striatal and ventral tegmental brain areas, specifically the nucleus accumbens (NAc) [11, 12]. Music listening may be rewarding, Menon and Levitin argue, because it mediates dopamine release, a neurotransmitter of reward, via the ventral tegmental-NAc network. Direct evidence of dopamine release to music has recently been found using positron emission tomography (PET) and functional magnetic resonance imaging (fMRI) [13].

Emotion regulation capabilities are determined in part by emotional biases in cognition. Individuals who have never had depression typically show a positivity bias in their cognitive processes [14]; they allocate greater attentional resources and show greater memory for positive versus negative information. While positive bias is typical, individuals with depression show an overall bias toward negative information. Depression is characterized by difficulties with emotion regulation [1], leading to prolonged negative affect and reduced responsiveness to previously enjoyed activities [15]. Negative affect may be the result of an increased susceptibility to negative emotional bias, including increased negative cognitions (attentional biases [16], outlook [17], rumination [15], and negative self-talk [18]), stemming from disordered brain activity [19]. Emotional responsiveness in depression is often altered for both positive and negative information. Individuals with MDD show reduced brain responses compared to control participants to emotionally positive words in reward centers of the brain, including ventral striatal and dorsomedial prefrontal regions [20]. Overall negativity biases in depression may comprise both negative hypersensitivity and positive hyposensitivity. Negative words are more readily processed by individuals with depression, as evidenced by shorter reaction times and larger evoked brain potentials, and responses to positive words are muted compared to control participants [21–23]. The negativity bias seen for emotional words may also occur in response to non-linguistic auditory information, such as music. If individuals with depression show a bias for negative musical stimuli—that is, increased brain responses or more negative ratings compared to control participants—it would be evidence that depression involves general hypersensitivity to negative information not limited to verbal rumination. Imaging studies have extended this negativity bias to music. Participants with MDD show reduced responses compared to control participants in ventromedial PFC (vmPFC) to their favorite music, despite similar enjoyment ratings between groups [24].While this dysregulation has been shown as an anhedonic response to favorite music [24], it has yet to be confirmed for potentially unfamiliar, emotionally evocative musical stimuli. By using standardized stimuli, familiarity effects and potential effects of participants’ self-awareness of loss of pleasure for familiar music are minimized.

The current research probes neural responsiveness to musical and nonmusical emotional stimuli that individuals are likely to encounter in their everyday lives. Emotional responses to music and other nonmusical stimuli have been studied separately in control participants, but though the patterns of responsiveness seem similar, the magnitude of these responses has not been directly compared for musical and nonmusical stimulus types. Individuals use music for emotion regulation [2]. Additionally, according to Thaut’s Rational Scientific Mediating Model of Music Therapy [25], the nonmusical goals of music therapy are achieved through the action of music on brain regions associated with nonmusical domains of functioning. Therefore, it is important to understand how underlying neural mechanisms may differ for these two stimulus types.

A large body of research has identified the anterior cingulate cortex (ACC) as a primary region implicated in major depressive disorder (MDD) [26]. [27]. The ACC is involved in both positive and negative emotional systems. The ACC receives projections from dopaminergic neurons in the ventral tegmental area and from top-down cognitive input in the prefrontal cortex [28]. It serves as a mediator between sensory inputs via the thalamus and appraisal via the prefrontal cortex, and is involved in monitoring for highly salient information [29–32]. Because of this, the ACC serves a critical function in task-switching, cognitive control, and emotional amplification and suppression [33]. While there is some disagreement on the number of discrete functional regions of the ACC, as well as the labeling of those regions [34–37], the ACC is often divided into two main areas: ventral (vACC) and dorsal (dACC). The vACC encompasses the perigenual, or rostral (rACC), and subgenual (sgACC) portions of the ACC. It is often described as the emotional ACC, because activation in this region is typically found to emotional stimuli [34]. Additionally, projections from the dopaminergic system reach this area of the ACC first. The dACC is defined posterior to the crossing of the corpus callosum to the motor cortex. It is described as the cognitive ACC, because it is more often activated in cognitive tasks that probe executive function and control, such as the Stroop task, which requires inhibition of prepotent responses [34, 35]. Volumetric studies repeatedly show decreased ACC size in participants with depression compared to control participants [38], including sufficient sensitivity and specificity to provide secondary means of diagnosis [39]. Unlike studies showing ongoing atrophy of affected brain regions with psychiatric disorders—for example, reduced hippocampal volume following PTSD [40]—the ACC in depression does not change size during the course of the disorder, nor with treatment [39]. This suggests that ACC volume may be a marker of depression vulnerability, in addition to its value in diagnosis. Functional responsiveness of the ACC has been linked to treatment success [27, 41]. Different treatment regimens target different subregions of the ACC; successful cognitive therapies are associated with increased activation in dorsal ACC, whereas medication based therapies often target ventral and rostral portions of the ACC [42]. Glutamate cycling, indicating general neural activity, has also been shown to be reduced in the vACC in depression [36], which suggests that tonic levels of activity in the vACC are lower in depression. For these reasons, we have focused on the ACC as the main region of interest in this study.

The current study uses fMRI and musical and nonmusical auditory-processing probes to determine whether activation within the ACC and striatum elicited by emotionally evocative auditory stimuli differ between the two stimulus types in never-depressed (ND) control participants. Further, this study measures differences in ACC and striatal activation to positive and negative emotional probes between participants with MDD and ND control participants, to confirm whether participants with depression also show negative bias for emotionally evocative non-linguistic auditory stimuli. We hypothesize that individuals with depression will show greater responsiveness to negative stimuli and reduced responsiveness to positive stimuli in ACC and striatum when compared with ND control participants.

## Methods and Materials

### Participants

#### ND participant population

This study was approved by the Human Subjects Committee of the University of Kansas Medical Center. Participants were recruited through email and flyer advertisements requesting volunteers with or without depression. All participants gave written consent prior to participation, according to the principles expressed in the Declaration of Helsinki. ND control participants (*n* = 22; 9 males; *M*_AGE_ = 28.50; *SD*_AGE_ = 11.14; *Range*_AGE_ = 18–59) were recruited with no history of depression or other psychiatric disorder, determined by administration of the Structured Clinical Interview for DSM Disorders, non-patient version (SCID-I/NP) [43]. Depression was assessed on the day of testing with the Beck Depression Inventory—Second Edition (BDI-II) [44], with a score greater than 18 indicative of high levels of depression. Based on this criterion, two participants scored greater than 18 on this measure on the day of testing and were excluded from further analyses (final group: *n* = 20).

#### MDD participant population

Twenty individuals with MDD (*n* = 20; 9 males; *M*_AGE_ = 34.15; *SD*_AGE_ = 13.64; *Range*_AGE_ = 18–56) were enrolled. Participants were all experiencing a current depressive episode at the time of scanning, determined by screening for research purposes using the SCID-I/NP [43]. Participants had no current or past manic episodes, no comorbid anxiety disorders, and no current alcohol abuse or dependence. One participant was taking medication for depression (Sertraline) at the time of the study, and was excluded from analyses; the final 19 participants were unmedicated. Five participants were currently undergoing counseling for depression, and 13 participants had received treatment in the past (Counseling: *n* = 7, Medication: *n* = 6). Behavioral treatments for depression, including counseling, have been shown to impact brain functioning [45, 46]; however, participants in this study were all experiencing a current depressive episode at the time of testing. Therefore, we believe measurements taken from this sample to be representative of the experience of clinically significant depressive symptoms that have not been ameliorated by the participants’ current or previous treatment. Four participants had a history of alcohol or drug dependence, fully remitted a minimum of one year prior to participation. One participant had a history of post-traumatic stress disorder (PTSD) in full remission. BDI-II scores were collected, but not used as criteria for inclusion/exclusion for the MDD group.

Participants in both groups were right-handed, had no contraindications for MRI (metal implanted in body, pregnancy), conditions and medications affecting blood flow (hypertension, diabetes), brain function (other psychiatric illness or medications), or neurological conditions (*e*.*g*., head injury, stroke). All participants had at least a high school education (*M*_ED_ = 15.22 years; *SD*_ED_ = 2.74 years), and were within normal or above average range of IQ (*M*_IQ =_ 118.95; *SD*_IQ =_ 11.65) as assessed by the Vocabulary and Matrix Reasoning subtests of the Wechsler Abbreviated Scale of Intelligence (WASI) [47]. The final groups (ND: *n =* 20, MDD: *n* = 19) did not significantly differ on age (*t*(37) = -1.02, *p* = .32, *M*_DIFF =_ -4.08, *SEM*_DIFF =_ 4.01), sex (*χ*^*2*^(1) = 0.03, *p* = .86), years of education (*t*(37) = 1.51, *p* = .14, *M*_DIFF =_ 1.32, *SEM*_DIFF =_ 0.87), IQ (*t*(37) = 1.16, *p* = .25, *M*_DIFF =_ 4.37, *SEM*_DIFF =_ 3.76), or years of musical training (*χ*^*2*^(4) = 1.80, *p* = .77).

### Materials

#### Stimuli

Emotionally evocative positive and negative musical examples from Western art music, and positive and negative nonmusical stimuli selected from the International Affective Digital Sound set (IADS) [48] were identified and validated through a separate rating study [49]. More than 400 10-second-duration audio clips from 12 pieces of Western art music were rated for emotional valence (positive-negative) and arousal (high-low) using the circumplex model of affect [50–52]. A final set of 36 musical (18 positive, 18 negative), 24 nonmusical (12 positive, 12 negative), and 9 neutral (pure tone) stimuli were used in this imaging study. These stimuli are fully described in a separate publication [49], and are available from the corresponding author (RL). In the previous rating study, positive and negative stimuli were rated significantly differently on valence. Additionally, musical and nonmusical stimuli were given comparable emotional valence and arousal ratings. Emotionally neutral pure tones were also identified and validated as intermediate between positive and negative stimuli. The three categories did not differ on arousal rating.

#### Questionnaires

Anxiety was assessed with the Beck Anxiety Inventory (BAI) [53]. This twenty-one item questionnaire assesses severity of anxiety symptoms over the previous week. The Affect Intensity Measure (AIM), a brief, 40-item validated self-report tool for measuring strength of positive and negative emotions, was collected to assess affect intensity [54]. Each item is rated on a six-point scale (Never—Almost Always). The AIM returns a total score from all items (*Range* = 40–240) and three subscale scores: Positive Affectivity (AIMPA; 15 items), Negative Intensity (AIMNI; 6 items), and Negative Reactivity (AIMNR; 6 items) [54, 55]. All participants underwent both SCID administration and BDI-II testing as part of this study. The SCID was used prior to enrolment to determine eligibility. The BDI-II, BAI, and AIM were collected on the day of fMRI testing as a measure of current mood and depressive symptoms, and those ND participants who scored high on the BDI-II were excluded as noted above.

### Procedures

#### fMRI methods

Participants underwent a single fMRI scanning session with anatomical scanning and five functional scanning runs. Scanning was conducted on a 3 Tesla Siemens Skyra scanner (Siemens, Erlangen, Germany). Participants’ heads were immobilized with cushions. Following automated scout image acquisition and shimming procedures to optimize field homogeneity, a structural scan was completed. High-resolution T1-weighted anatomic images were acquired with a 3D MPRAGE sequence (TR/TE = 2300/2.01 msec, flip angle = 9°, FOV = 256 mm, matrix = 256x192, slice thickness = 1 mm), used for slice localization for the functional scans, Talairach transformation, and coregistration with fMRI data. Participants were given the option to have their de-identified structural images included in a database accessible to researchers at the institution, reducing the cost of future studies. Following structural scans, five gradient echo blood oxygen level dependent (BOLD) sequences were acquired in 50 interleaved oblique axial slices at a 40° angle (repetition time/echo time [TR/TE] = 3000/25 msec, flip angle = 90°, field of view [FOV] = 220 mm, matrix = 64x64, slice thickness = 3 mm, 0 mm skip, in-plane resolution = 2.9x2.9 mm, 105 data points, 5 min: 24 sec).

To minimize susceptibility artifact and optimize signal in ventromedial prefrontal regions, participants were positioned in the scanner with the angle of the AC-PC plane between 17° and 22° in scanner coordinate space, verified with a localization scan. This careful positioning ensured that the 40° slice acquisition angle was applied the same way for all subjects. Head positioning and slice orientation parameters were verified in pilot tests and are now applied routinely at the imaging center in all fMRI studies targeting ventromedial regions of the brain.

**Fig 1:**
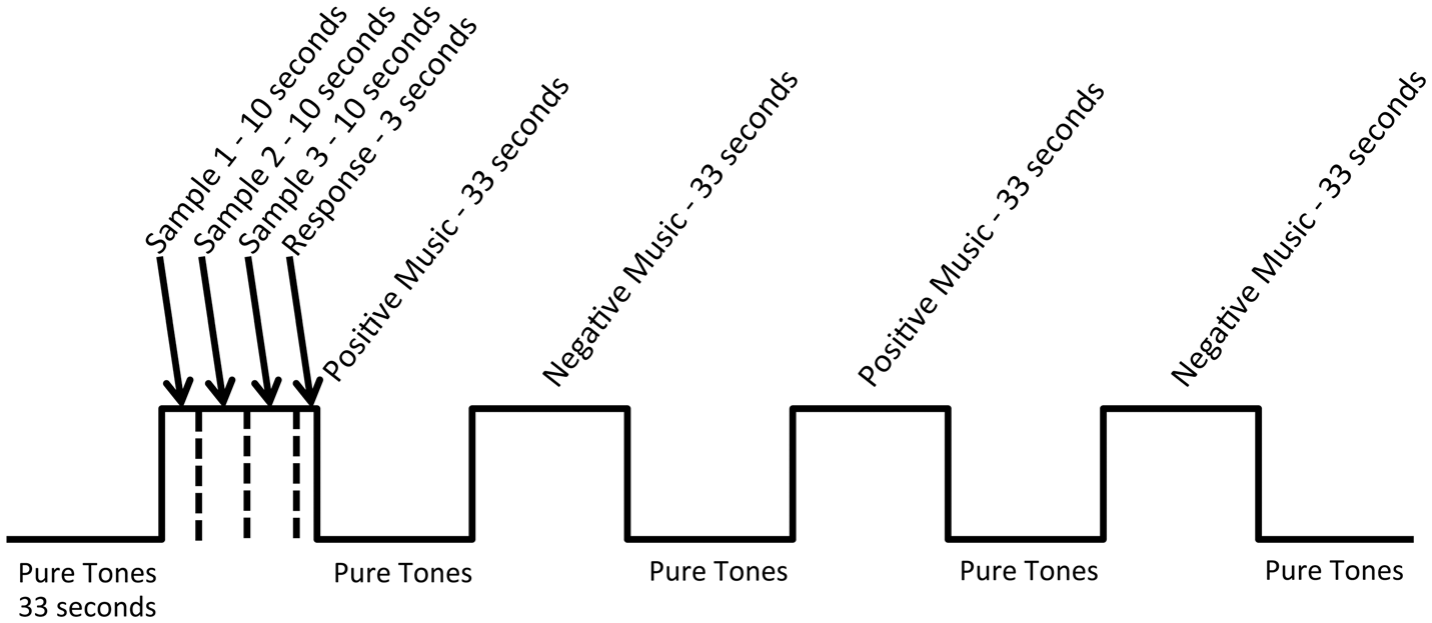
fMRI Paradigm. An example functional run from the blocked emotional stimulus paradigm.

During the functional runs, auditory stimuli were presented in blocks of three clips from the same experimental condition (Fig 1) using E-Prime 2.0 software (Psychology Software Tools, Inc., Sharpsburg, PA), with six stimuli from each experimental condition presented during each run. To ensure that participants were attending to the emotional content of the stimuli, they were instructed to think about whether each clip was emotionally positive or negative while listening, and after each block of clips, were asked to rate whether the preceding block as a whole consisted of positive or negative stimuli. During three functional runs, participants listened to alternating groups of positive music, negative music, and pure tones. During the remaining two functional runs, participants listened to alternating groups of positive and negative nonmusical stimuli (IADS) [48] and pure tones. Pure tones were used as the baseline to control for general auditory stimulation and pitch, while neither being musical nor nonmusical. This stimulus condition was validated as emotionally neutral compared to the musical and nonmusical stimuli in the previously published rating study [49]. Stimuli were presented through MR compatible earbuds (Sensimetrics Corporation, Malden, MA) at 70dB, or as loud as comfortably possible to ensure the stimuli were heard over the noise of the scanner. Volume levels were adjusted for each subject with an audio test in the scanner prior to the task. Participants heard music not included in the task [56] concurrent with the noise of a functional BOLD sequence and provided visual feedback (thumbs up or down) to indicate whether the volume should be raised or lowered. In addition, noise-canceling headphones were placed over the earbuds to block scanner noise. This system was designed to present audio stimuli against the noise of the MR environment and has been used successfully during fMRI scanning.

#### Emotion rating methods

Using methods developed in our previous ratings studies [49], participants gave valence and arousal ratings for the stimuli following fMRI scanning. Ratings were collected after scanning to ensure that the stimuli were novel during scanning. Auditory stimuli were presented through computer speakers, using E-Prime 2.0 software running on a PC computer outside the scanning environment. Participants were allowed to adjust the volume to a comfortable level during a practice session. After each stimulus, participants were presented with a biaxial diagram, with valence rating coded on the *x* axis, and arousal coded on the *y* axis [49]. To encourage participants to rate their experience of emotion, rather than emotions that they simply recognized, participants were explicitly instructed to rate how each stimulus made them feel. Responses were collected via mouse click, and the mouse position in pixels was recorded for both *x* and *y* (*Origin* (*x* = 314, *y* = 240); *RangeX* = 76 (left)–542 (right); *RangeY* = 16 (upper)–464 (lower), resulting in a single valence and arousal rating per 10-second stimulus per subject.). The procedure lasted approximately twenty minutes. Following the rating procedure, participants completed questionnaires and were debriefed.

### Analysis

#### Questionnaires

Group differences (MDD, ND) on each of the self-report measures were assessed using two-sample *t*-tests.

#### Emotion ratings

Average valence and arousal ratings for each condition (Valence—Positive, Negative: Type—Musical, Nonmusical) given by participants in the two diagnosis Groups (MDD, ND), were compared using separate 2x2x2 mixed model analysis of covariance (ANCOVA) tests to determine whether the groups were responding to the two stimulus types differently. Gender, age, and years of musical training were included as covariates in each analysis. Planned analyses using one-tailed *t*-tests directly tested whether MDD participants rated the negative stimuli as more negative and the positive stimuli as less positive compared to ND participants by comparing average ratings of valence across the diagnostic groups. Demographics, summary questionnaire scores, and emotion ratings for each participant are provided in Supporting Information S1 File.

#### fMRI preprocessing

fMRI data were analyzed using the Analysis of Functional NeuroImages (AFNI) statistical package [57]. Preprocessing steps included trilinear 3D motion correction, 3D spatial smoothing to 4 mm with a Gaussian filter, and high pass filter temporal smoothing. Images were resampled to a voxel-wise resolution of 2.5 mm^3^. Each participant’s structural image was realigned to the first functional image obtained within the participant’s scanning session, and normalized to the space defined by Talairach and Tournoux’s stereotaxic atlas [58] with the AFNI <@auto-tlrc> algorithm. Normalization to atlas space was confirmed by visual inspection for all participants. Anatomic data from one participant (MDD group) could not be successfully normalized using the <@auto-tlrc> algorithm, and was transformed manually in AFNI by defining key anatomic points (anterior commissure, posterior commissure, anterior point, posterior point, superior point, inferior point, right point, left point, and two points on the mid-sagittal plane). Volumes with excessive signal artifact (>50% voxels considered outliers were censored from each dataset prior to statistical analyses. Additionally, motion of greater than 1 mm between successive TRs resulted in the censoring of that TR and the two adjacent TRs. No functional runs were discarded for excessive motion (*i*.*e*.>30%).

#### fMRI statistical analyses

**Fig 2:**
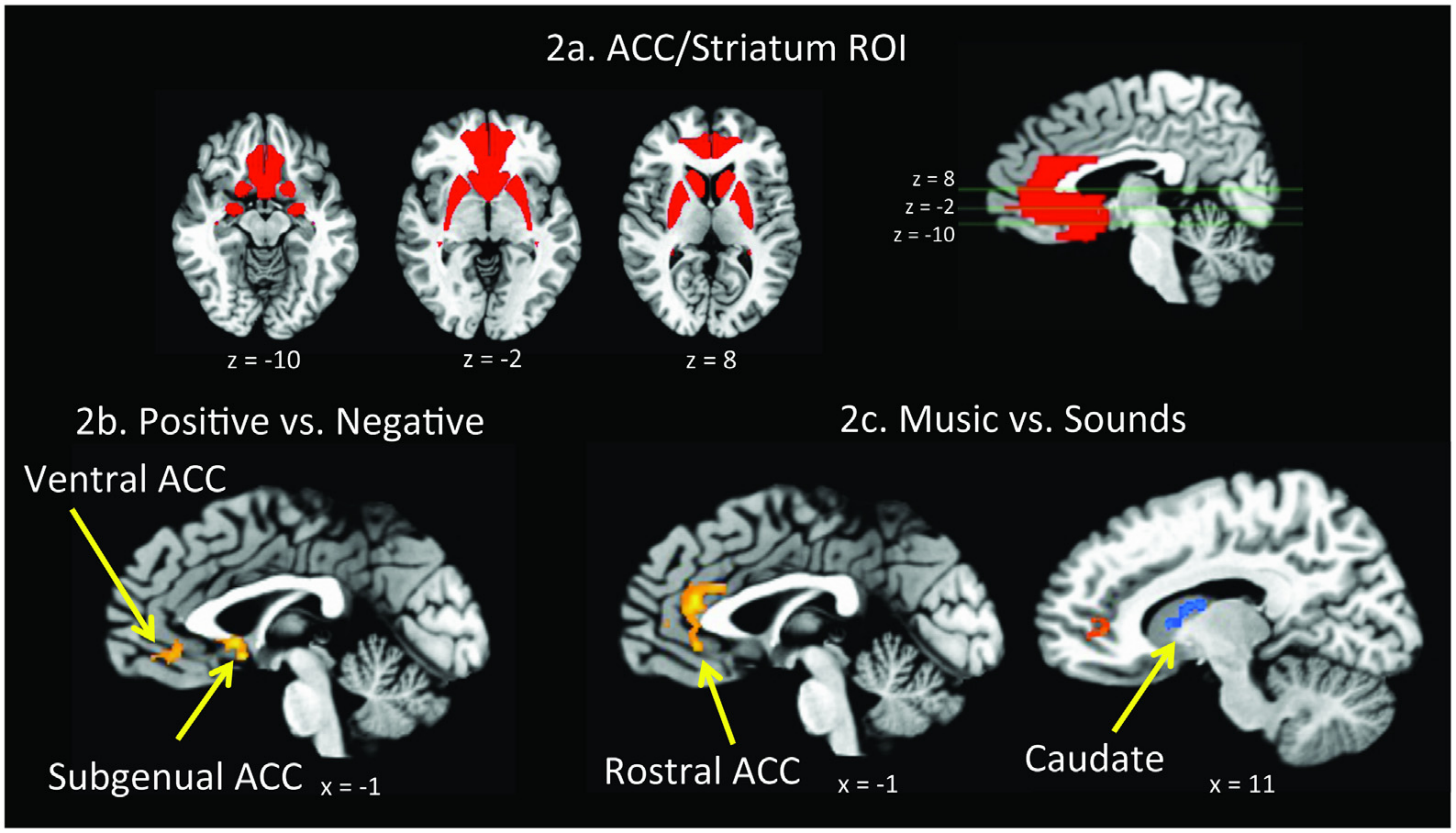
Region of Interest and fMRI Results—ND Participants. 2a. Anterior cingulate cortex and striatum region of interest (ACC/striatum ROI). 2b. Among ND participants, Ventral and Subgenual regions of the ACC exhibited significantly greater activation to positive versus negative stimuli. 2c. Among ND participants, Rostral ACC showed significantly greater activation to musical versus nonmusical stimuli, while right Caudate showed significantly greater activation to nonmusical versus musical stimuli.

Activation maps were analyzed using statistical parametric methods [59] contained within the AFNI software [57]. Statistical contrasts were conducted using multiple regression analysis with the general linear model (GLM). Regressors representing the experimental conditions of interest were modeled with a hemodynamic response filter and entered into multiple regression analysis using a two-stage mixed-effects model. Motion estimates were also entered into the model as nuisance regressors. Contrasts between conditions of interest were assessed with *t* statistics using the <3dttest++> command. We conducted whole brain exploratory analyses and a region of interest (ROI) analysis focused on regions implicated in emotion processing in depression, including ACC (Brodmann areas (BA) 32 and 33, sgACC (BA25), and striatum (amygdala, caudate, nucleus accumbens, and putamen), defined anatomically from Talairach template masks within AFNI and combined into a single ACC/striatum mask (Fig 2A). Main effects of Valence (Positive, Negative) and Stimulus Type (Musical, Nonmusical), and the interaction of Valence and Stimulus Type were assessed over the whole brain in the ND group, and also in the ACC/striatum ROI mask to confirm activation to the task within the ACC and striatum. Whole brain and ROI comparisons were also conducted between ND and MDD groups to determine whether depression status interacted with Valence or Stimulus Type within these critical regions. Statistical parametric maps were overlaid on three-dimensional renderings of the Talairach template brain (TT_N27). Activations were considered significant if they survived a statistical threshold of *p*_corrected_ <.01 in the whole brain analyses, or *p*_corrected_ <.05 within the ACC/striatum ROI mask (small volume corrected for multiple comparisons determined by Monte Carlo simulations using AFNI’s <3dClustSim> command).

## Results and Discussion

### Questionnaires

Descriptive statistics and results of the two-sample *t*-tests are provided in Table 1. Participants with depression reported higher total BDI-II scores and total BAI scores, confirming that this group was experiencing greater depressive symptoms at the time of testing, as well as higher anxiety. Participants with depression also had significantly higher scores on the negative intensity (AIMNI) subscale of the AIM, as well as somewhat higher scores, though not significant, on the negative reactivity (AIMNR) subscale, indicating that they generally experience negative situations with greater intensity, and may have greater reactivity to negative situations, than do ND participants.

**Table 1:** Comparisons of Questionnaire Scores between MDD* and ND^†^ participants.

Score	*t*	*Df*	*p*	*M(SD) MDD*	*M(SD) ND*	*M*_DIFF_	*SEM*_DIFF_
BDI-II‡	-10.78**	37	<.001	30.16 (10.63)	3.00 (3.67)	-27.16	2.52
BAI§	-7.46**	37	<.001	10.09 (10.09)	2.65 (3.36)	-17.77	2.38
AIM¶ Total	-1.35	37	.19	143.11 (23.08)	134.25 (17.73)	-8.86	6.57
AIM Positive Affectivity	0.43	37	.67	52.00 (13.76)	53.55 (8.48)	1.55	3.64
AIM Negative Intensity	-4.25**	37	<.001	22.63 (6.12)	15.50 (4.24)	-7.13	1.68
AIM Negative Reactivity	-1.65	37	.11	24.47 (4.10)	22.45 (3.56)	-2.02	1.23

*Major depressive disorder,

^†^Never depressed,

^‡^Beck Depression Inventory, 2^nd^ Edition,

** significant a *p* <.01,

^§^Beck Anxiety Inventory,

^¶^Affect Intensity Measure.

### Emotion ratings

The results of the ANCOVA for Valence rating revealed a significant main effect for Valence (*F*(1, 34) = 9.45, *p =* .004, η^2^ = .22), with positive stimuli being rated as more positive than negative stimuli (*M*_POS_ = 357.96, *SEM*_POS_ = 3.87; *M*_NEG_ = 269.51, *SEM*_NEG_ = 4.49). No other effects or interactions passed significance criteria.

There was a trend toward a significant interaction of Valence by Type (*F*(1, 34) = 3.10, *p =* .09, η^2^ = .08), with positive music being rated somewhat more positive than positive nonmusical stimuli (*M*_DIFF_ = -42.81, *M*_POS_MUS_ = 379.36, *SEM*_POS_MUS_ = 4.84; *M*_POS_NONMUS_ = 336.55, *SEM*_POS_NONMUS_ = 6.38), and negative music being significantly more positive as negative nonmusical stimuli but with a smaller mean difference (*M*_DIFF_ = -20.79, *M*_NEG_MUS_ = 279.91, *SEM*_NEG_MUS_ = 6.62; *M*_NEG_NONMUS_ = 259.12, *SEM*_NEG_NONMUS_ = 5.93). Finally, there was a trend toward a significant interaction of Valence by Type by Age (*F*(1, 34) = 3.21, *p =* .08, η^2^ = .09). Though not significant, this three-way interaction was characterized by older participants (> = 27 years) rating negative nonmusical stimuli as marginally more negative than did younger participants (*t*(37) = -1.87, *p* = .07, *M*_DIFF_ = -21.19, SEM_DIFF_ = 11.36), but no difference was found for positive nonmusical stimuli (*t*(37) = -1.40, p = .17, *M*_DIFF_ = -18.12, *SEM*_DIFF_ = 12.91), positive music (t(37) = 1.05, *p* = .30, *M*_DIFF_ = 10.54, *SEM*_DIFF_ = 10.02) or negative music (*t*(37) = -0.50, *p* = .62, *M*_DIFF_ = -6.64, *SEM*_DIFF_ = 13.18).

There was not a significant interaction of Valence by Group (*F*(1, 34) = 0.80, *p =* .38, η^2^ = .02), which means that diagnostic group was not a factor in how the participants were rating the valence of these stimuli. The planned *t*-tests comparing Valence ratings of positive (*t*(37) = 0.32, *p* = .75, *M*_DIFF_ = 2.46, *SEM*_DIFF_ = 7.79) and negative stimuli (*t*(37) = 1.16, *p* = .25, *M*_DIFF_ = 10.50, *SEM*_DIFF_ = 9.03) between the two groups were non-significant, confirming this result. All other effects and interaction terms in the ANCOVA were non-significant (All *F*’s < 2.5).

The results of the ANCOVA for Arousal rating revealed no significant effects. However, there was a trend toward a significant interaction of Valence by Group (*F*(1, 34) = 3.51, *p =* .07, η^2^ = .09), with ND participants rating positive stimuli as slightly more arousing than MDD participants (*M*_DIFF_ = 17.21, *SEM*_DIFF_ = 10.60, *M*_*ND_*POS_ = 224.67, *SEM*_*ND_*POS_ = 8.12, *M*_*MDD_*POS_ = 207.46, *SEM*_*MDD_*POS_ = 6.72), and no difference for negative stimuli (*M*_DIFF_ = -3.35, *SEM*_DIFF_ = 9.77, *M*_*ND_*NEG_ = 223.70, *SEM*_*ND_*NEG_ = 7.44, *M*_*MDD_*NEG_ = 227.05, *SEM*_*MDD_*NEG_ = 6.26). There was also a trend toward a significant interaction of Type by Age (*F*(1, 34) = 3.57, *p =* .07, η^2^ = .10), with younger participants (< 27 years) rating nonmusical stimuli as more arousing than did older participants (*t*(37) = -2.66, *p* = .01, *M*_DIFF_ = -31.45, *SEM*_DIFF_ = 11.83), but no difference was found for musical stimuli (*t*(37) = -0.29, *p* = .77, *M*_DIFF_ = -3.42, *SEM*_DIFF_ = 11.75). There was also a trend for the main effect of Age (*F*(1, 34) = 3.00, *p =* .09, η^2^ = .08). Though not significant, the pattern of arousal ratings indicated that younger participants rated all stimuli as slightly more arousing than did older participants (*M*_<27_ = 230.20, *SEM*_<27_ = 6.36; *M*_> = 27_ = 212.76, *SEM*_> = 27_ = 5.48). All other effects and interaction terms were non-significant (All *F*’s < 2.5).

As there were no significant main effects or interactions of diagnostic group for valence or arousal rating, these null findings suggest the two groups did not differ in their subjective responses to the stimuli. Therefore, differences in subjective labeling of the stimuli cannot be the explanation for the fMRI results that follow.

### ND Participants: whole brain fMRI results

#### Main effect of Valence

When all positive stimuli were compared to all negative stimuli, the right auditory cortex showed significantly greater activation to positive stimuli, while bilateral occipital gyri showed significantly greater activation to negative stimuli. There was significantly greater activation to positive stimuli in ventral anterior cingulate and right hippocampus. Activation focused in the hippocampus also spread into the right dorsal amygdala (Table 2).

**Table 2:** Whole-brain activations to the emotional auditory functional MRI task, Main effect of Valence—ND Participants.

Contrast and Region	*X*	*Y*	*Z*	*Peak t statistic*	*Cluster size (mm*^*3*^*)*
***ND***‡**: *Main effect of Valence; Positive > Negative***					
Right Auditory cortex	54	-4	1	6.20	2891
vACC§	-4	36	-9	5.10	2172
Right Hippocampus/Amygdala	26	-11	-9	4.69	1641
Right Superior Temporal cortex	59	-19	9	4.38	1313
***ND*: *Main effect of Valence; Negative > Positive***					
Right Occipital cortex	24	-86	9	-4.88	2766
Left Occipital cortex	-31	-81	6	-5.36	1797

Coordinates for the maximally activated voxel are provided in Talairach space. Correction for multiple comparisons, whole-brain corrected *p* < .01; *t* > 2.09, cluster >1125 mm^3^).

^‡^Never depressed,

^§^Ventral anterior cingulate cortex.

#### Main effect of Stimulus Type

In stark contrast to the focal Valence results, the main effect of stimulus type revealed broad differences in activation across the brain. Activation foci are listed in Table 3; however, clusters were large and encompassed several regions. Activation to musical stimuli was stronger in bilateral middle frontal gyrus, anterior and dorsal cingulate gyrus, bilateral precuneus, bilateral inferior parietal lobule, bilateral occipital gyrus, and bilateral fusiform gyrus. Activation to nonmusical stimuli was greater in thalamus, striatum, dorsomedial prefrontal cortex (DMPFC), bilateral dorsolateral prefrontal cortex (DLPFC), bilateral ventrolateral PFC (VLPFC), auditory cortex bilaterally (middle temporal gyrus, superior temporal gyrus), and bilateral cerebellum.

**Table 3:** Whole-brain activations to the emotional auditory functional MRI task, Main effect of Stimulus Type—ND participants.

Contrast and Region	*X*	*Y*	*Z*	*Peak t statistic*	*Cluster size (mm*^*3*^*)*
***Main effect of Stimulus Type; Musical > Nonmusical***					
Left (and Right) Precuneus/Parietal/dACC§	-26	-56	51	6.27	63266
Right Inferior Temporal/Occipital/Fusiform Gyri	49	-64	-1	6.09	13641
Left Middle Occipital/Fusiform Gyri	-49	-76	6	5.27	9766
Right Middle Frontal Gyrus	26	26	34	4.39	1609
Left Middle Frontal Gyrus	-21	34	34	5.73	1516
Left Insula	-39	9	9	5.15	1469
Right Cerebellum	19	-49	-51	5.32	1234
***Main effect of Sound Type; Nonmusical > Musical***					
Right (and Left) Cerebellum	21	-64	-29	-8.55	23344
Right Auditory cortex	46	-16	6	-7.23	18328
Left Auditory cortex	-56	-46	16	-6.93	14500
Left DLPFC¶/VLPFC**	-39	14	29	-6.76	14156
Right DLPFC/VLPFC	36	24	21	-6.12	13797
Left (and Right) Thalamus/Striatum	-6	-11	11	-6.23	7813
Right DMPFC††	1	11	59	-6.74	4859
Left Striatum	-21	-11	-4	-6.43	4594
Right Parahippocampus/Amygdala	26	-6	-9	-6.01	2734
Right Cerebellum	9	-46	-29	-5.25	2438
Left DMPFC	-4	41	36	-4.17	1438

Coordinates for the maximally activated voxel are provided in Talairach space. Correction for multiple comparisons, whole-brain corrected *p* < .01; *t* > 2.09, cluster >1125 mm^3^).

^§^Dorsal anterior cingulate cortex,

^¶^Dorsolateral prefrontal cortex,

**Ventrolateral prefrontal cortex,

^††^Dorsomedial prefrontal cortex.

#### Interaction of Valence by Stimulus Type

In the interaction of Valence by Stimulus Type, bilateral auditory cortex and bilateral precentral gyrus showed significant activation characterized by greater activation to positive music versus negative music, with no difference in activation to positive versus negative nonmusical stimuli. Right inferior frontal cortex had greater activation to negative versus positive music, with no difference for positive versus negative nonmusical stimuli. Right parietal cortex had significantly greater activation for negative versus positive music, and greater activation for positive versus negative nonmusical stimuli (Table 4).

**Table 4:** Whole-brain activations to the emotional auditory functional MRI task, Interaction of Valence by Stimulus Type—ND Participants.

Contrast and Region	*X*	*Y*	*Z*	*Peak t statistic*	*Cluster size (mm*^*3*^*)*
***ND***‡**: *Positive > Negative; Musical > Nonmusical***					
Left Auditory cortex	-59	-16	11	7.48	13172
Right Auditory cortex	49	-6	4	5.65	12891
Left Precentral Gyrus	-49	-11	41	4.39	1406
Right Precentral Gyrus	44	-9	44	4.53	1344
***ND*: *Negative > Positive; Musical > Nonmusical***					
Right Parietal cortex	36	-59	41	-3.61	1734
Right Inferior Frontal Gyrus	51	19	14	-4.04	1391

Coordinates for the maximally activated voxel are provided in Talairach space. Correction for multiple comparisons, whole-brain corrected *p* < .01; *t* > 2.09, cluster >1125 mm^3^).

^‡^Never depressed.

### ND ACC ROI fMRI results

Within the ACC/striatum mask, there was a significant main effect of Valence, with greater activation to positive versus negative stimuli in vACC (*p*_corrected_ <.01), and sgACC (*p*_corrected_ <.04) (Fig 2B). There was also a main effect of Stimulus Type in rACC, with greater activation for musical compared to nonmusical stimuli (*p*_corrected_ <.001), and in right caudate with greater activation for nonmusical stimuli compared to music (*p*_corrected_ <.02; Fig 2C; Table 5).

**Table 5:** Significant activations within the ACC*/Striatum ROI^†^ –ND participants.

Contrast and Region	*X*	*Y*	*Z*	*Peak t statistic*	*Cluster size (mm*^*3*^*)*
***ND***‡**: *Main effect of Valence; Positive > Negative***					
vACC§	-4	36	-9	5.10	1391
sgACC¶	1	6	-6	5.41	656
***ND*: *Main effect of Valence; Negative > Positive***					
NS**					
***ND*: *Main effect of Sound Type; Musical > Nonmusical***					
rACC††	-1	34	19	5.44	3641
***ND*: *Main effect of Sound Type; Nonmusical > Musical***					
Right Caudate	11	11	6	-4.41	500

Coordinates for the maximally activated voxel are provided in Talairach space. Correction for multiple comparisons, small volume corrected *p* < .05; *t* > 2.09, cluster > 375 mm^3^).

*Anterior cingulate cortex,

^†^Region of Interest,

^‡^Never depressed,

^§^Ventral anterior cingulate cortex,

^¶^Subgenual anterior cingulate cortex,

**No significant clusters,

^††^Rostral anterior cingulate cortex.

### Comparison of MDD and ND groups: whole brain fMRI results

No areas of the cortex were significantly activated in the Group by Valence interaction (all *p*_corrected_ > .05).

Comparing group responses to musical versus nonmusical stimuli (Interaction of Group by Stimulus Type), significant activations were found in the anterior cingulate and the dorsolateral prefrontal cortex (Table 6).

**Table 6:** Activations to the emotional auditory functional MRI task, Group Interactions.

Contrast and Region	*X*	*Y*	*Z*	*Peak t statistic*	*Cluster size (mm*^*3*^*)*
***Group by Valence Interaction; ND***‡ ***> MDD***‡‡**: *Positive > Negative***					
NS**					
***Group by Valence Interaction; MDD > ND*: *Positive > Negative***					
NS**					
***Group by Stimulus Type Interaction; ND > MDD*: *Musical > Nonmusical***					
dACC§§	-1	31	14	4.22	1672
Left DLPFC¶	-21	46	26	3.92	1531
***Group by Stimulus Type Interaction; MDD > ND*: *Musical > Nonmusical***					
NS					
***Group by Valence by Stimulus Type Interaction***					
NS					

Coordinates for the maximally activated voxel are provided in Talairach space. Correction for multiple comparisons, whole-brain corrected *p* < .01; *t* > 2.03, cluster > 1125 mm^3^)

^‡^Never depressed,

^‡‡^Major depressive disorder,

*No significant clusters,

^§§^Dorsal anterior cingulate cortex,

^¶^Dorsolateral prefrontal cortex.

No areas of the cortex were significantly activated in the three-way Group by Valence by Stimulus Type interaction (all *p*_corrected_ > .05). One small cluster in the left caudate tail showed a trend toward a significant interaction (*p*_corrected_ > .06; [TAL XYZ = -26–46 4] 906 mm^3^). In this region, ND participants showed greater activation for all positive versus all negative stimuli, whereas MDD participants showed greater activation for positive versus negative music, and greater activation for negative versus positive nonmusical stimuli.

### Comparison of MDD and ND groups: ACC ROI fMRI results

#### Interaction of Group by Valence

**Fig 3:**
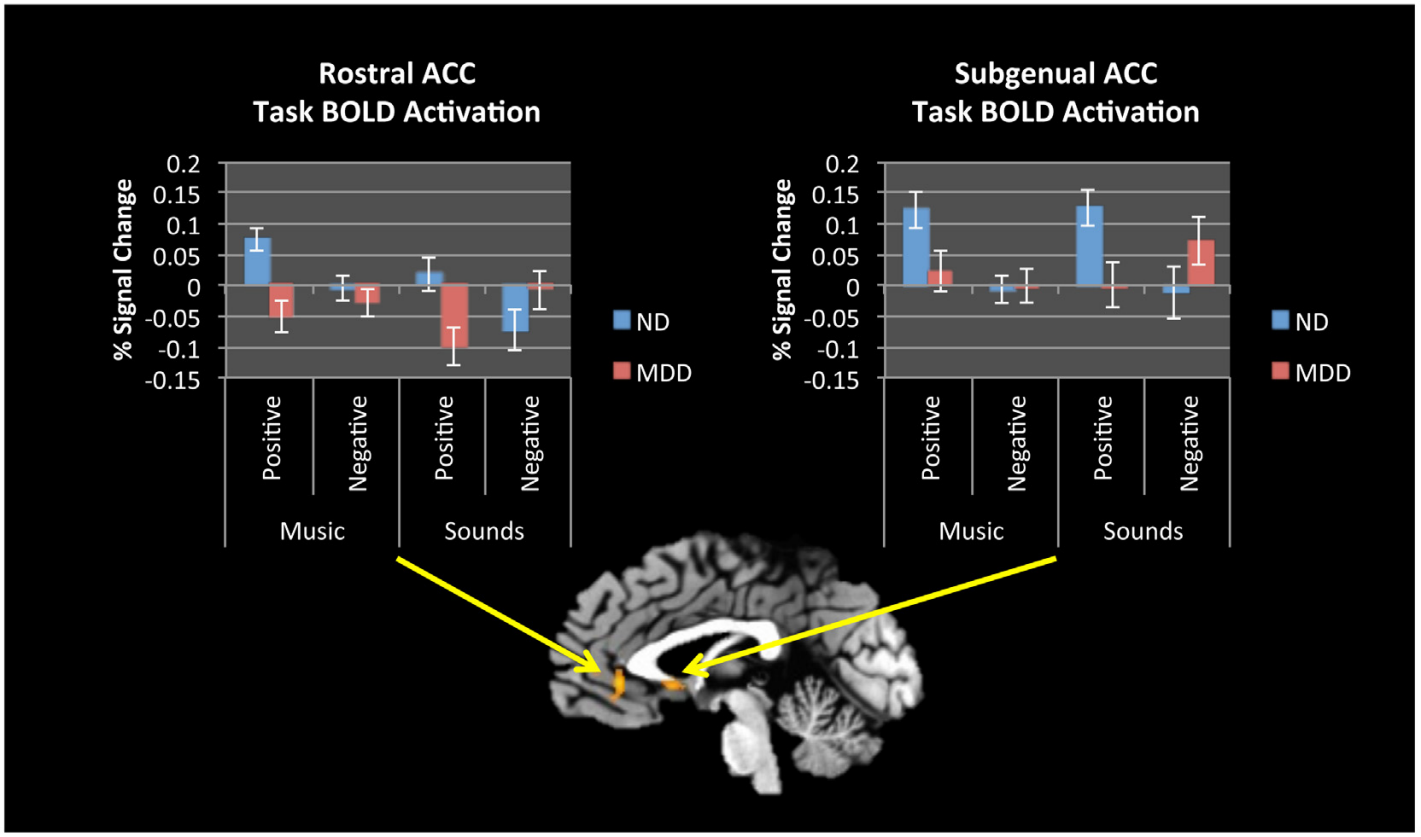
fMRI Results—Group by Valence. Rostral and Subgenual ACC showed differential task activation between ND and MDD groups to positive versus negative stimuli. Graphs show mean activation over the entire cluster. Error bars denote standard error.

Comparing all positive to all negative stimuli, significant differences were found between MDD and ND groups in rACC (*p*_corrected_ <.01), with ND participants increasing activation from baseline to positive stimuli and decreasing activation from baseline to negative stimuli, whereas participants with depression showed no difference from baseline to negative stimuli and a significant decrease to positive stimuli (Fig 3; Table 7). There was also a significant group difference in sgACC (*p*_corrected_ <.03), with ND participants increasing activation from baseline for positive stimuli with no difference from baseline to negative stimuli, whereas participants with depression exhibited the opposite pattern. No significant group differences were found in striatum.

**Table 7:** Significant activations within the ACC*/Striatum ROI^†^ –Group comparison.

Contrast and Region	*X*	*Y*	*Z*	*Peak t statistic*	*Cluster size (mm*^*3*^*)*
***Group by Valence Interaction; ND***‡ ***> MDD***‡‡**: *Positive > Negative***					
rACC††	-1	34	1	3.92	625
sgACC¶	1	11	-1	4.00	484
***Group by Valence Interaction; MDD > ND*: *Positive > Negative***					
NS**					
***Group by Stimulus Type Interaction; ND > MDD*: *Musical > Nonmusical***					
dACC§§	-1	31	14	4.22	656
***Group by Stimulus Type Interaction; MDD > ND*: *Musical > Nonmusical***					
NS					
***Group by Valence by Stimulus Type Interaction***					
vACC§ (*p* <.09)	-9	36	-6	-4.64	359

Coordinates for the maximally activated voxel are provided in Talairach space. Correction for multiple comparisons, small volume corrected *p* < .05; *t* > 2.03, cluster > 375 mm^3^).

*Anterior cingulate cortex,

^†^Region of Interest,

^‡^Never depressed,

^‡‡^Major depressive disorder,

^††^Rostral anterior cingulate cortex,

^¶^Subgenual anterior cingulate cortex,

**No significant clusters,

^§§^Dorsal anterior cingulate cortex,

^§^Ventral anterior cingulate cortex.

#### Interaction of Group by Stimulus Type

**Fig 4:**
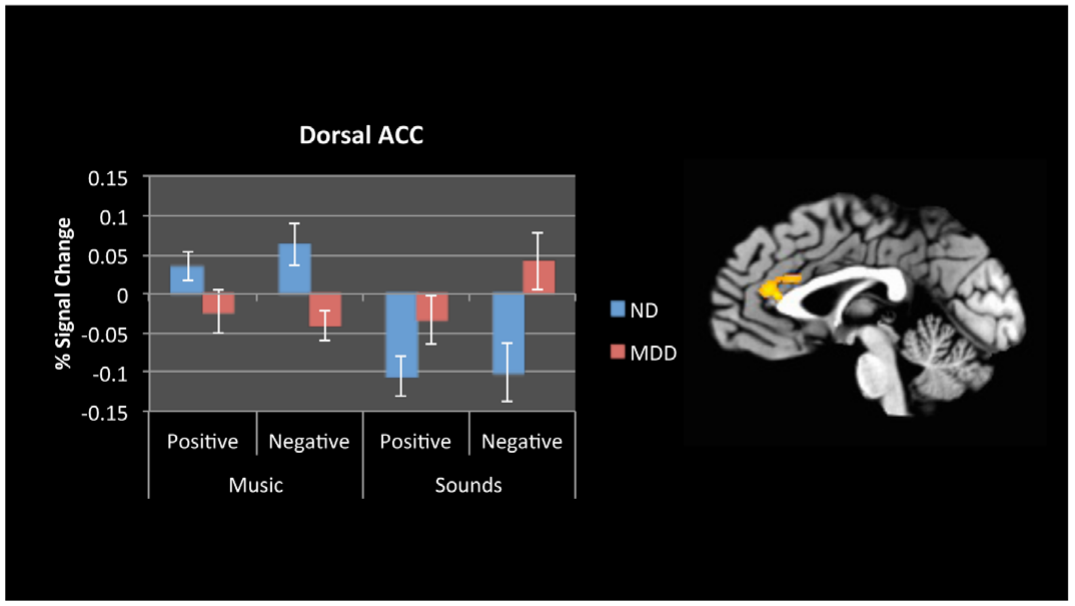
fMRI Results—Group by Stimulus Type. Dorsal ACC showed differential task activation between ND and MDD groups to musical versus nonmusical stimuli. Graphs show mean activation over the entire cluster. Error bars denote standard error.

Comparing group responses to musical versus nonmusical stimuli, only one region survived thresholding (*p*_corrected_ <.01). ND participants had greater activation in dACC to musical versus nonmusical stimuli, whereas participants with depression had greater activation to nonmusical stimuli compared to music, with relatively greater activation to negative nonmusical stimuli, less activation to positive nonmusical stimuli, then positive music, and finally, negative music (Fig 4; Table 7). The area of activation covers both vACC and dACC; however, the maximally activated voxel was in dACC.

#### Three-way interaction

There was a trend toward a significant cluster in vACC in the Group by Valence by Stimulus Type interaction (*p*_corrected_ <.09; Table 7). In this region, both ND and MDD participants had greater activation to positive versus negative music; however, ND participants had greater activation to positive versus negative nonmusical stimuli, whereas MDD participants had greater activation to negative versus positive nonmusical stimuli.

## Discussion

This experiment was designed as a probe for investigating neural circuitry of emotion and reward in depression. First, the paradigm was tested in ND control participants to determine whether musical and nonmusical stimuli activated these circuits. After confirming the paradigm activated a priori defined ACC and striatal regions, activation was directly compared within the anatomic mask with an unmedicated group of participants experiencing a current depressive episode. Both groups reported similar emotional experiences from the stimuli; however, the ACC showed differences in activation. No group differences were observed in striatum.

### ND control participants

#### Positive versus Negative stimuli

Positive stimuli activated vACC to a greater extent than negative stimuli for both groups. Activation in this region was characterized by a pure valence effect—no differences were seen based on stimulus type. The vACC receives dopamine projections from the ventral tegmental area, and sends projections dorsally and laterally to executive control areas of the cortex [28]. Blunted activation in this region has been associated both with transient sadness [35] and with depression [26], suggesting that this region is critical for the experience of positive emotions. These findings corroborate previous research [11, 13] and suggest that the dopaminergic system is active during music listening. While familiarity has been shown to impact both liking [60] as well as neural responsiveness for music [61], we have no reason to believe that familiarity would differ for the positive and negative stimuli presented in this study. We chose to use Western art music examples, rather than popular or film music, to minimize familiarity effects. The dopaminergic system was also activated by nonmusical stimuli in this study, as evidenced by greater activation in caudate to nonmusical stimuli versus music. The musical and nonmusical stimuli used in this study were equally emotional [49]. However, the nonmusical stimuli were concrete, nameable items or experiences, whereas the music was abstract. It is possible that another feature, such as self-referential memory, may have driven activation in the caudate, but this is speculative. Further work is needed to elucidate the differential roles of ACC and striatum in processing emotional stimuli of various types.

#### Musical versus Nonmusical stimuli

The responses to musical versus nonmusical stimuli were described by very different patterns of activation. Greater activation to nonmusical compared to musical stimuli in thalamus, amygdala, cerebellum and auditory cortex—regions associated with early emotion processing—suggests that nonmusical stimuli activate primary emotion networks more than music. However, greater activation to nonmusical stimuli was also found in lateral and dorsomedial prefrontal cortex, areas associated with top-down executive control, object recognition, language processing, and reappraisal. Music, on the other hand, activated rostral anterior cingulate cortex, precuneus, and bilateral parietal and occipital cortices more than nonmusical stimuli. These regions, collectively, are associated with the default mode network (DMN), a network of regions that tend to be more active when a person is focused more on their own internal state, rather than engaged in an external task [62, 63]. These two systems have been described in models of voluntary and automatic reappraisal strategies [64]. The DMN has been implicated in autobiographical processing [65, 66]. The fact that music activates the DMN, while nonmusical stimuli show greater activation in the network generally associated with tasks of executive function, is neural evidence that supports Myer’s [67] theory that the ambiguity in music is what allows it to be what he called a “metaphorizing medium,” a scaffold that provides structure, but not content, that allows individual listeners the freedom to impose personal meaning onto this structure.

These stimuli were matched for valence and arousal [49]; therefore, the differences seen here cannot be attributed to differences in the emotional qualities of the stimuli, and must, therefore, be interpreted in terms of cognitive identification and appraisal strategies. Together, these findings show that even when carefully matched for emotional content, separable brain networks process music compared with other emotional sounds. Emotional nonmusical stimuli activate early emotion monitoring systems (thalamus, amygdala, and cerebellum). Executive control areas, such as DLPFC, VLPFC, and DMPFC are activated as well, suggesting that object identification and voluntary reappraisal may be taking place. Music activates DMN and reward processing areas, such as ACC, suggesting that emotional processing in music relies more on autobiographical memory, idiosyncratic meaning assignment, and automatic appraisal, than does emotional processing in everyday sounds, which are more concrete and activate linguistic processing areas to a greater degree.

### Comparison of MDD and ND groups

#### Positive versus Negative stimuli

When all stimuli were compared based on emotional valence, vACC (rACC and sgACC) showed relatively more activation to positive stimuli in ND participants. In rACC, this relative difference was driven by ND participants showing increased activation to positive stimuli, with a significant decrease from baseline to negative stimuli. Participants with depression showed the opposite pattern: no difference from baseline for negative stimuli, but a significant decrease from baseline to positive stimuli. In sgACC, ND participants had increased activation for positive stimuli and no difference from baseline to negative stimuli, whereas participants with depression had increased activation for negative stimuli and no difference from baseline to positive stimuli. This represents both a hypoactivation to positive and a hyperactivation to negative stimuli among depressed participants. The sgACC has been shown to be the most effective stimulation site for deep brain stimulation in treatment-resistant depression [68]. Although the activation found in this study extends beyond the sgACC, the entire ACC receives projections from the midbrain dopaminergic neurons (ventral tegmental areas) and is implicated in emotional functioning in depression [69–71]. Again, familiarity could have an impact on these results [61]; however, while we did not measure familiarity directly in this study, we did measure musical training—which did not differ between groups. For these reasons, we feel confident that our results reflect differential emotional processing between the groups, rather than familiarity.

The current findings using standardized emotional auditory stimuli replicate those found by Osuch and colleagues [24], who showed that participants with depression had reduced activation to their favorite music in this region. Here, we show that decreased reactivity to positive stimuli in depression can generalize to evocative emotional stimuli, including music and positive nonmusical stimuli, and that this effect extends to other subregions of ACC. The vACC inhibits amygdala response [33], and has been shown to deactivate during cognitive tasks [34]. Activation patterns observed in this region could indicate monitoring for a change from one’s current mood state, as ND participants showed a change in activation only to negative stimuli and participants with depression showed a change only to positive stimuli.

#### Musical versus Nonmusical stimuli

By matching for arousal, we were also able to directly compare responses to music and nonmusical stimuli, further extending the work from Osuch and colleagues [24]. Comparing musical to nonmusical stimuli between the groups, activation was found in perigenual and dorsal ACC. In this region, ND participants showed greater activation to all music compared to all nonmusical stimuli. Participants with depression showed greater activation to nonmusical stimuli in this region, with the biggest response for negative nonmusical stimuli, smaller response for positive music, then positive nonmusical stimuli, and negative music showing the smallest response. Even sad music can be enjoyable and aid in emotion regulation [72–77]. When emotional content is mild—as in this study—and stimuli are not dissonant or designed to be unpleasant yet evoke negative emotions—as in the negative music condition—emotional classification for the music requires a decision between competing streams of information.

#### Emotion ratings

Self-reported emotion ratings for Valence and Arousal indicate that depression status did not systematically influence how participants rated the stimuli. In the current study, enjoyment and pleasure were not directly measured, however, the lack of group differences in subjective experience is comparable to the findings of Osuch and colleagues [24]. In that study, participants gave equivalent enjoyment ratings for their favorite music, yet showed reduced activation in reward centers of the brain, suggesting a potential neural marker of anhedonia.

### Implications

The current study sought to determine whether emotional musical and nonmusical stimuli were processed similarly by healthy participants. Specifically, we had hypothesized that negative music and nonmusical stimuli might be processed differently, given the growing body of literature focused on the enjoyment of sad music [75–77]. In addition, the study compared the evoked brain responses to those stimuli in people with and without MDD to determine if the pattern of response was affected by MDD. By comparing musical and nonmusical stimuli, the current study provides a broader understanding of how individuals with MDD process different types of auditory stimuli. Music is currently used for mood manipulations in clinical and laboratory settings [5–10]; therefore, the results may ultimately have significant clinical implications for treating depression, or for the use of music as an affective probe for determining risk of developing the disorder. Additionally, music therapists have been using music to impact mood and depression in terminal illness [78] and Alzheimer’s disease [79], and are now extending this to mood disorders that are not related to a physical illness, with promising results [80–83]. The transitory nature of music might make it a useful tool for mood modification; however, the mechanisms by which this may occur are not fully defined. Koelsch and colleagues [81] argue that music therapy may be useful in treating depression, PTSD, and other mood disorders by acting on both the NAc-VTA reward processing loop, and by potentially reactivating the anterior hippocampal formation, which has been shown to have a reduced volume in these disorders [84]. The current results suggest that, similar to other forms of treatment, the mechanisms by which music and other forms of emotional auditory stimulation may function in depression could be by reactivating the ACC. As the link between ACC and emotion regulation has also been established in other psychiatric conditions such as borderline personality disorder [85–87], the present results may also have implications for psychiatric conditions beyond depression.

### Limitations

Although the stimuli used in this study were carefully matched for both valence and arousal, the measure used to assess emotional ratings was based on self-report. While participants were explicitly instructed to rate how the stimuli made them feel, this self-report style measure did not allow for examination of whether the emotion was truly experienced by participants or simply recognized. Also, the fMRI employed a block-design, which did not allow us to probe responses to individual stimuli, or exclude trials to which participants may have been responding differently than expected. Future studies should include psychophysiological measurements, such as heart rate variability, respiration, and skin conductance, and further fMRI studies could use event-related designs that would allow individual variability in response to be measured. Additionally, a limited number of examples from Western art music are used in the current study. An increase in the number and variety of musical examples would be beneficial. Though these examples were selected empirically to control for familiarity and linguistic confounds, other genres of music, such as popular songs or opera, might elicit stronger emotions. Additionally, although there is evidence to suggest that many of the emotional cues in music, such as harmonic expectancies, are learned through enculturation rather than explicit musical training [88], and that emotional responses to music are influenced by familiarity [61], there is a possibility that brain responses could differ based on musical preference or training. While the lack of a familiarity measure is a limitation of this study, we did measure musical training. In this sample of participants musical training ranged from none (*n* = 8) to more than ten years (*n* = 7); however, the two groups were matched for years of musical training, thereby limiting the confounding effect of training across group.

## Conclusions

In conclusion, the present project revealed that in healthy participants, positive auditory stimuli activated reward-processing areas of the brain that are implicated in depression. This set of studies focused on the ACC, which showed differential responsiveness to these mild emotional stimuli in participants with depression, and striatum. By using fMRI and a standardized set of musical and nonmusical emotion-processing probes, the current study provides insight into finer distinctions of stimulus type and may have implications for therapeutic interventions or risk assessment. The pattern of responsiveness in the ACC among participants with depression in this study raises the question of whether music, and specifically positive music, may be useful in retraining the ACC and improving functioning. A longitudinal study with a music-listening intervention would be critical to determine whether activation in ACC is malleable in this population. It is also possible that both the initial emotional response to a stimulus and the inability to sustain activation of positive emotional neural circuitry may lead to persistent depression, as reported by Heller and colleagues [89]. Results from this and other studies of affective responsivity in MDD may lead to more effective and targeted treatments.

## Supporting Information

S1 File Demographics, summary questionnaire scores, and emotion ratings by participant. Key: study_id = unique subject identifier; male (1 = male, 0 = female); age (years), ed = education in years; mus_train (1 = None, 2 = 1–3 years, 3 = 4–6 years, 4 = 7–10 years, 5 = more than 10 years); MDD = Major Depressive Disorder classification (1 = MDD group, 0 = Never Depressed group); AIMtot = Affect Intensity Measure total score; AIM_PA = AIM positive affectivity subscore; AIM_NI = AIM negative intensity subsore; AIM_NR = AIM negative reactivity subscore; BDI_Tot = Beck Depression Inventory total score; BAI_Tot = Beck Anxiety Inventory total score; WASI_iq = estimated IQ from two subtests of Wechsler Abbreviated Scale of Intelligence; NegNonMus_X = average valence rating for negative nonmusical stimuli; NegNonMus_Y = average arousal rating for negative nonmusical stimuli; NegMus_X = average valence rating for negative musical stimuli; NegMus_Y = average arousal rating for negative musical stimuli; PosNonMus_X = average valence rating for positive nonmusical stimuli; PosNonMus_Y = average arousal rating for positive nonmusical stimuli; PosMus_X = average valence rating for positive musical stimuli; PosMus_Y = average arousal rating for positive musical stimuli.
(CSV)
