# Correction: Neural Processing of Emotional Musical and Nonmusical Stimuli in Depression

**DOI:** 10.1371/journal.pone.0163631

**Published:** 2016-09-20

**Authors:** Rebecca J. Lepping, Ruth Ann Atchley, Evangelia G. Chrysikou, Laura E. Martin, Alicia A. Clair, Rick E. Ingram, W. Kyle Simmons, Cary R. Savage

The third author’s name is spelled incorrectly. The correct name is: Evangelia G. Chrysikou. The correct citation is: Lepping RJ, Atchley RA, Chrysikou EG, Martin LE, Clair AA, Ingram RE, et al. (2016) Neural Processing of Emotional Musical and Nonmusical Stimuli in Depression. PLoS ONE 11(6): e0156859. doi:10.1371/journal.pone.0156859
